# Adrenalin for Sensitivity

**Published:** 1904-12

**Authors:** W. Kay

**Affiliations:** Stomatologist.


ADRENALIN FOR SENSITIVETY.—Apply adrelin chloride, 1-000, on a pellet of cotton for a few moments. Remove and place full strength formalin on another pellet of cotton to which apply a suitable-sized *hot* ball-burnisher. After about half a minute the most sensitive dentine can be comfortably excavated. Of course, care must be taken to protect the patient's eyes and air-passages from the formadehyde fumes.—*Dr. W. Kay, Stomatologist.*

